# Development and application of an AllGlo probe-based qPCR assay for detecting knockdown resistance (*kdr*) mutations in *Anopheles sinensis*

**DOI:** 10.1186/1475-2875-13-379

**Published:** 2014-09-23

**Authors:** Liang Bai, Guo-ding Zhu, Hua-yun Zhou, Jian-xia Tang, Ju-lin Li, Sui Xu, Mei-hua Zhang, Li-nong Yao, Guang-quan Huang, Yong-bin Wang, Hong-wei Zhang, Si-bao Wang, Jun Cao, Qi Gao

**Affiliations:** Key Laboratory of Parasitic Disease Control and Prevention (Ministry of Health), and Jiangsu Provincial Key Laboratory of Parasite Molecular Biology, Jiangsu Institute of Parasitic Diseases, Wuxi, Jiangsu Province People’s Republic of China; Institute of Plant Physiology & Ecology, Shanghai Institute for Biological Sciences, Chinese Academy of Sciences, Shanghai, People’s Republic of China; Department of Parasitology, Medical College of Soochow University, Suzhou, 215123 People’s Republic of China; Zhejiang Center for Disease Control and Prevention, Hangzhou, People’s Republic of China; Hubei Center for Disease Control and Prevention, Wuhan, People’s Republic of China; Shandong Institute of Parasitic Diseases, Jining, People’s Republic of China; Henan Center for Disease Control and Prevention, Zhengzhou, People’s Republic of China; Public Health Research Center, Jiangnan University, Wuxi, People’s Republic of China

**Keywords:** *Anopheles sinensis*, Knockdown resistance (*kdr*), AllGlo probe, qPCR

## Abstract

**Background:**

*Anopheles sinensis* is one of the most important malaria vectors in China and other Southeast Asian countries. High levels of resistance have been reported in this species due to the long-term use of insecticides, especially pyrethroids, for public health and agricultural purposes. Knockdown resistance (*kdr*) caused by a single base pair mutation in the gene encoding the sodium channel is strongly associated with pyrethroid insecticide resistance in many *Anopheles* mosquitoes. There are few methods currently available for detecting *kdr* mutations in *An. sinensis*.

**Methods:**

A novel AllGlo probe-based qPCR (AllGlo-qPCR) method was developed to screen for the predominant *kdr* mutations in *An. sinensis* mosquitoes from the Jiangsu Province. The results from AllGlo-qPCR, allele-specific PCR (AS-PCR), and *TaqMan*-MGB probe-based qPCR (*TaqMan*-qPCR) were compared. A comparative analysis of the equipment required, ease of use and cost of the available methods was also performed. Finally, the AllGlo-qPCR method was used to detect the frequencies of *kdr* mutations from the other four provinces in central China.

**Results:**

Six *kdr* genotypes were detected in *An. sinensis* from the Jiangsu Province by DNA sequencing. The AllGlo-qPCR method detected all of the *kdr* genotypes with a high level of accuracy (97% sensitivity and 98% specificity). AllGlo-qPCR correctly determined the *kdr* genotypes of 98.73% of 158 *An. sinensis* samples, whereas *TaqMan*-qPCR and AS-PCR correctly identified 96.84% and 88.61% of mutations, respectively. Furthermore, the AllGlo-qPCR method is simpler to perform, requires less equipment, and exhibits a moderate expense cost comparing with the other tested methods of *kdr* mutation detection. Samples collected from four of the other provinces in central China showed a high frequency of *kdr* mutation in *An. sinensis*, as detected by the established AllGlo-qPCR method.

**Conclusion:**

The novel AllGlo-qPCR method developed for *kdr* mutation detection in *An. sinensis* exhibits greater specificity and sensitivity than currently available methods and is more cost-effective; therefore, it represents a useful tool for entomological surveillance.

## Background

*Anopheles sinensis* is an important member of the *Anopheles hyrcanus* group [1]. It is widely distributed in China, Korea, Japan and other Asian countries and is a major malaria vector, especially in central China [2]. There is increasing interest in this species due to its high abundance and modest susceptibility to malarial parasites [3, 4].

Vector control remains the primary component of malaria control strategies, even for countries that are in the malaria elimination phase [5]. Currently, the most accepted methods for treatment involve using the insecticide pyrethroid for indoor residual spraying (IRS) and insecticide-treated nets (ITNs) in the regions with a high risk of malarial transmission [6]. However, the large-scale use of pyrethroid for public health and agricultural purposes has resulted in the rapid spread of resistance in malaria vectors. Reports of pyrethroid resistance in *An. sinensis* have increased dramatically in recent years [7, 8]. Urgent action is needed to prevent the emergence of resistance at new sites and to maintain the effectiveness of vector control interventions in the short, medium and long-term. The latest global plan for insecticide resistance management in malaria vectors (GPIRM) released by the World Health Organization (WHO) advocates collecting baseline data associated with insecticide resistance in major vectors to strengthen scientific collaboration and to further understand the mechanisms of insecticide resistance at both global and national levels [9].

Pyrethroids continuously activate voltage-gated sodium channels (VGSCs) in insects, leading to spasms, paralysis and death. However, amino acid substitutions in segment 6 of domain II of the VGSC result in insensitivity to pyrethroids. Reduced sodium channel target-site sensitivity is a major mechanism of pyrethroid resistance and is referred to as knockdown resistance (*kdr*) [10]. Many previous studies have demonstrated that the *kdr* is strongly associated with insecticide resistance in *Anopheles* mosquitoes, including *An. sinensis* [7, 11, 12].

There are currently very few assays available to screen for DNA substitutions that lead to *kdr* mutations in *An. sinensis*. The most commonly used method is allele-specific PCR (AS-PCR) [7, 13] (also known as competitive PCR amplification of a specific allele (cPASA) [11]), due to its relatively low cost. However, this technique can generate inaccurate results due to mismatches at the 3’ end of the primer, and if the system is poorly optimized, it can be difficult to determine band brightness. To improve screening accuracy, a *TaqMan*-MGB probe-based assay was developed to detect *kdr* mutations [14]. Unfortunately, this approach is unable to identify all *kdr* mutation genotypes within a single reaction: two parallel reaction tubes are required to provide complete mutation information. In this study, a novel AllGlo probe-based screening method was developed to overcome these problems. This method is based on sequencing a portion of the sequence coding for VGSC in *An. sinensis*. Using this method, the frequencies of *kdr* mutations in several provinces in central China were detected.

## Methods

### Mosquito collection and identification

**Figure 1:**
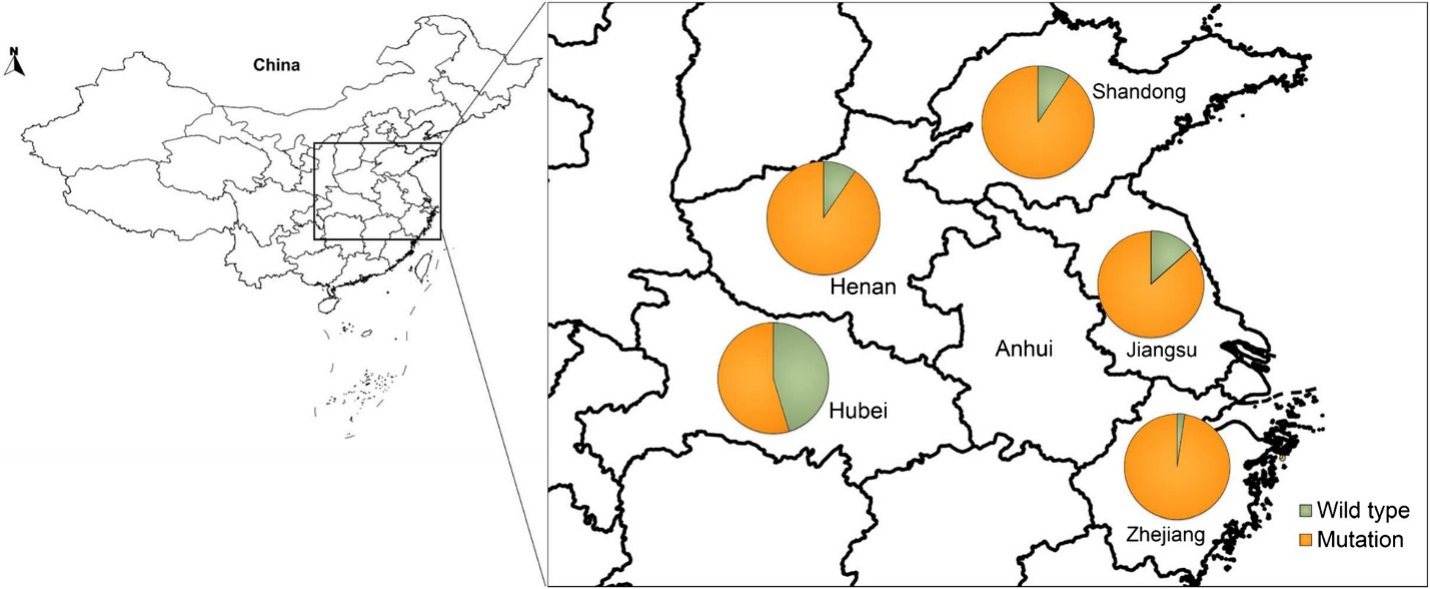
**The collection sites for** ***An. sinensis*** **in central China.**

Wild anopheline mosquitoes were collected from vector surveillance sites in Jiangsu Province in 2011 and were transferred to the insectary of the Key Laboratory on Technology for Parasitic Disease Control and Prevention, Ministry of Health, Jiangsu Institute of Parasitic Diseases (JIPD) in Wuxi, Jiangsu Province, China. Wild *An. sinensis* mosquitoes were also collected from the other provinces in central China, including Zhejiang, Henan, Shandong and Hubei (Figure 1). The wild mosquito species were identified by rDNA-ITS2 [15], and only those confirmed as *An. sinensis* were selected for the following studies. The laboratory colonies of *An. sinensis* have been cultured in JIPD for more than thirty years without exposure to any insecticides.

### gDNA extraction

Total genomic DNA was extracted from each individual mosquito using a Fast Tissue-to-PCR kit (Thermo scientific) according to the manufacturer’s instructions. Briefly, one or two mosquito legs were added to a mixture of 100 μL of Tissue Lysis Solution and 10 μL of Proteinase K and were incubated at 55°C for 10 min and 95°C for 10 min. Next, 100 μL of Neutralization Solution T was added to the mixture, which was then centrifuged at 17,900 × g for 3 min. The supernatant was transferred to a new tube and stored at −30°C for species identification and *kdr* detection.

### Nucleotide sequencing

The *kdr* genotypes of individual mosquitoes were determined by DNA sequencing. A partial segment of the VGSC gene that included the *kdr* mutations was amplified from laboratory *An. sinensis* strains and randomly selected wild *An. sinensis* strains from the Jiangsu Province using the previously described primers *kdr*-F (5’-TGCCACTCCGTGTGTTTAGA-3’) and *kdr*-R 5’-GAGCGATGATGATCCGAAAT-3’) [7]. The PCR products were electrophoresed on a 1.5% agarose gel containing 0.5 μg/mL ethidium bromide. Direct PCR sequencing of both strands was performed by Genscript (Nanjing, China).

### Development of the AllGlo-qPCR method

**Figure 2:**
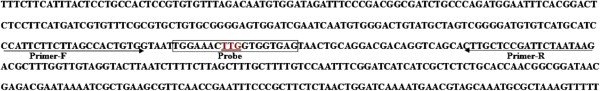
**Schematic representation of the primers and probes used to detect** ***kdr*** **mutations in** ***An. sinensis*** **.** Arrows represent the forward primer (left) and the reverse primer (right). The box represents the location where the probe used to detect wild-type (red) and mutated genotypes anneals.

The nucleotide sequences generated as described above were aligned and compared to the available sequence in NCBI (GenBank NO: GI84646709), and the conserved region surrounding the *kdr* site was selected for primer/probe design. We designed forward and reverse primers and 3 AllGlo probes based on the sequenced *kdr* genotypes (Shanghai Huirui Biotechnology Co., Ltd). The forward primer (primer-F: 5’-CCATTCTTCTTAGCCACTGTG-3’) and the reverse primer (primer-R: 5’-CTTATTAGAATCGGAGCAA-3’) were standard oligonucleotides with no modifications. The *kdr*-TTG probe (MAR-TGAAACTTGGTGGTGAG-MAR) for detection of the wild-type allele was labelled with MAR at the 5’ end, the *kdr*-TTT probe (JUP-TGGAAACTTTGTGGTGA G-JUP) for detection of *kdr*-TTT was labelled with JUP and the *kdr*-TGT probe (NEP-TGAAACTGTGTGGTGGAG-NEP) for detecting *kdr*-TGT was labelled with NEP (Figure 2). The wild-type and mutated *kdr* genotypes, TTG, TTT and TGT, were detected using a Roche Lightcycler480 via the FAM channel (465 nm-510 nm), the VIC/HEX channel (533 nm-580 nm) and the CY5 channel (618 nm-660 nm), respectively. The 10 μL PCR reactions contained 1 μL of genomic template, 5 μL of 2× reaction master mix which contains 2× reaction buffer, dNTPs, Taq DNA polymerase, Mg^2+^ and RNaseH, 0.5 μL of 10 μM each primer, 0.4 μL of 10 μM each probe, and distilled water to 10 μL. PCR amplification was performed on a Roche Lightcycler480, using the following conditions: 5 minutes at 95°C, followed by 40 cycles of 95°C for 15 seconds and 60°C for 30 seconds. The changes in FAM, VIC/HEX, and CY5 fluorescence were monitored in real time by acquiring the signal at 60°C for each cycle.

### Analysing the accuracy of AllGlo-qPCR compared to AS-PCR and *TaqMan*-qPCR

To verify the accuracy of the AllGlo-qPCR method, the *kdr* genotypes of both the field (108) and laboratory (50) samples were simultaneously detected using the AllGlo-qPCR method and two previously established approaches, AS-PCR and *TaqMan-*qPCR.

### Comparative analysis of AllGlo-qPCR, AS-PCR and *TaqMan*-qPCR

The special equipment required, protocol run time, number of steps, primers/probes required, number of reaction tubes and the average cost per sample (excluding the cost of equipment and machine maintenance) were compared between AllGlo-qPCR, AS-PCR, *TaqMan*-qPCR and DNA sequencing.

### Detecting *kdr*mutation frequencies in samples collected from central China

The *kdr* mutation frequency was investigated in samples from the other provinces in central China, including the Henan, Hubei, Zhejiang and Shandong Provinces, using the AllGlo-qPCR method established above.

## Results

### Predominant *kdr*mutations in *An. sinensis*

Multiple mutated *kdr* genotypes were identified via sequencing of 108 wild and 50 laboratory *An. sinensis* mosquitoes. All of the 50 laboratory samples were homozygous for the wild-type sequence (TTG/TTG). By contrast, 5 *kdr* genotypes were detected in the wild samples, including the dominant homozygotic genotype TTT/TTT in 61 mosquitoes, the heterozygotic genotype TTT/TGT in 30 mosquitoes, and low percentages of TTG/TTT, TTG/TGT and TGT/TGT genotypes (found in 3, 1 and 1 mosquitoes, respectively). In addition, 12 wild *An. sinensis* mosquitoes with the wild-type genotype (TTG/TTG) were identified.

### AllGlo-qPCR-based detection of *kdr*mutations

**Figure 3:**
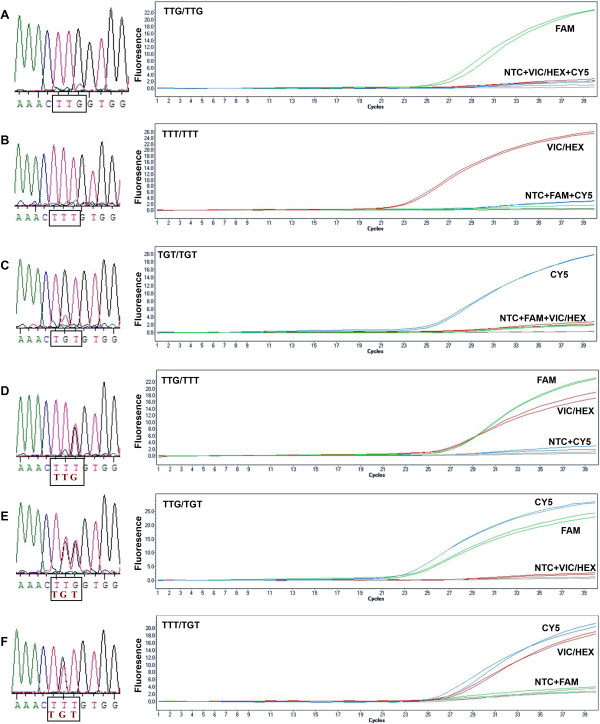
**Comparison of DNA sequencing and AllGlo-qPCR amplification of TTG, TTT and TGT in** ***An. sinensis*** **.** The panels on the left show the sequencing results, and the panels on the right show the AllGlo-qPCR amplification curves. **A**: TTG homozygote; **B**: TTT homozygote; **C**: TGT homozygote; **D**: TTG/TTT heterozygote; **E**: TTT/TGT heterozygote; **F**: TTG/TGT heterozygote; NTC: no template control.

**Figure 4:**
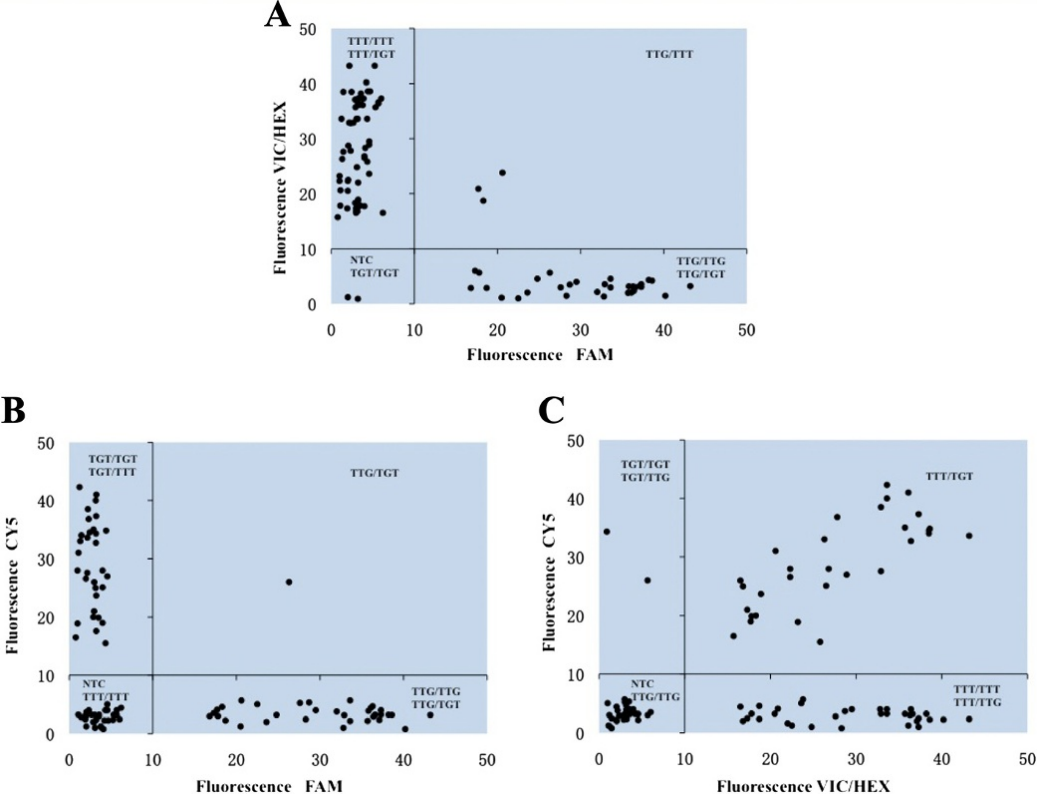
**Scatter plot analysis of** ***kdr*** **mutations in** ***An. sinensis*** **detected by AllGlo-qPCR.** Endpoint fluorescence intensities between **(A)** FAM and VIC/HEX; **(B)** FAM and CY5; **(C)** VIC/HEX and CY5. PCR was performed in 96-well plates. The FAM, VIC/HEX and CY5 fluorescence intensities were measured and corrected for background and then plotted in a bi-directional scatter plot using Microsoft Excel. NTC: no template control.

The AllGlo-qPCR assay detected the six known *kdr* genotypes in *An. sinensis* and was 100% consistent with sequencing results. Three probes were added simultaneously to a single reaction. A substantial increase in FAM fluorescence indicated a wild-type homozygote, and a substantial increase in HEX/VIC or CY5 fluorescence indicated a homozygous mutant. An intermediate increase in any of the two signals indicated heterozygosity (Figure 3). The *kdr* genotypes were scored with software, and the endpoint fluorescence intensities between any two dyes were plotted against each other on bi-directional scatter plots (Figure 4). All six of the known *kdr* genotypes could be identified easily in comparison to the three bi-directed clusters.

### AllGlo-qPCR detected *kdr*mutations with greater sensitivity and specificity than current methods

The 158 samples with known *kdr* genotypes were tested by AS-PCR, *TaqMan*-MGB and AllGlo-qPCR methods. A comparison of the results showed that *TaqMan*-qPCR and AllGlo-qPCR were more sensitive and specific than AS-PCR, as both methods demonstrated 100% sensitivity with only one exception (discrimination of TTT/TTT) and 100% specificity with only one exception (discrimination of TTT/TGT) out of the six total *kdr* genotypes. However, the AS-PCR method generated a relatively higher level of incorrect results, leading to lower sensitivity and specificity (Table 1). In general, the AllGlo-qPCR method was most accurate (98.73%), followed by *TaqMan*-MGB (96.84%) and AS-PCR (88.61%) when compared to nucleotide sequencing (Table 1).

**Table 1:** **Comparison of** ***kdr*** **mutations in** ***An. sinensis*** **detected by AS-PCR,** ***TaqMan*** **-qPCR and AllGlo-qPCR versus direct sequencing**

Genotype	Detection assay*				Sensitivity (95% confidence level)			Specificity (95% confidence level)		
	Sequencing	AS-PCR	***TaqMan***- qPCR	AllGlo-qPCR	AS-PCR	***TaqMan***- qPCR	AllGlo-qPCR	AS-PCR	***TaqMan***- qPCR	AllGlo-qPCR
TTG/TTG	62	59	62	62	95% (87%,98%)	100% (94%,100%)	100% (94%,100%)	100% (96%,100%)	100% (96%,100%)	100% (96%,100%)
TTT/TTT	61	60^a^	56	59	89% (78%,94%)	92% (82%,96%)	97% (89%,99%)	94% (87%,97%)	100% (96%,100%)	100% (96%,100%)
TGT/TGT	1	2^b^	1	1	100% (21%,100%)	100% (21%,100%)	100% (21%,100%)	99% (96%,99.9%)	100% (98%,100%)	100% (98%,100%)
TTG/TTT	3	5^c^	3	3	67% (21%,94%)	100% (44%,100%)	100% (44%,100%)	98% (94%,99%)	100% (98%,100%)	100% (98%,100%)
TTG/TGT	1	2^d^	1	1	100% (21%,100%)	100% (21%,100%)	100% (21%,100%)	99% (96%,99.9%)	100% (98%,100%)	100% (98%,100%)
TTT/TGT	30	30^e^	35^f^	32^g^	77% (59%,88%)	100% (89%,100%)	100% (89%,100%)	95% (89%,97%)	96% (91%,98%)	98% (94%,99.6%)
Total	158	158	158	158						
Accuracy**	-	88.61%	96.84%	98.73%						

*:The superscript letter indicates an incorrect result generated by AS-PCR, *TaqMan*-qPCR or AllGlo-qPCR, compared to standard sequencing, as follows: a: 6 TTT/TGT; b: 1 TTT/TGT; c: 3 TTG/TTG; d: 1 TTG/TTT; e: 7 TTT/TTT; f: 5 TTT/TTT; g: 2 TTT/TTT.

**:The accuracy refers to the percentage of correct results generated by AS-PCR, *TaqMan*-qPCR or AllGlo-qPCR out of 158 known *kdr* genotype samples.

### AllGlo-qPCR is easier to use with moderate cost

The equipment required, ease of use and the cost of four available methods for detecting *kdr* mutations in *An. sinensis* were summarized (Table 2). More specialized equipment and more steps are needed for sequencing and AS-PCR, whereas only a real-time PCR machine and one experimental step are required for the other two methods. In addition, the *TaqMan*-MGB and AllGlo-qPCR methods do not entail the use of hazardous chemicals, such as ethidium bromide solution, and the run time (less than 2 h) is much shorter than sequencing and AS-PCR. Sequencing was the most expensive technique, and AS-PCR was the least expensive. AllGlo-qPCR was less expensive than *TaqMan*-MGB, as only a single reaction tube was needed rather than two.

**Table 2:** **Comparison of four** ***kdr*** **genotyping assays based on the need for specialized equipment, cost, safety, simplicity and speed**

Method	Equipment required	Hazardous chemicals	Protocol run time	No. of steps	Primers/probe required	No. of tubes required per sample	Cost per run*
Sequencing	PCR thermocycler	Ethidium bromide	6 h	2	2 PCR primers	1	$4.20
	Gel electrophoresis and imaging equipment						
	Nucleic acid sequencing machine						
AS-PCR	PCR thermocycler	Ethidium bromide	4 h 30 min	2	5 PCR primers	2	$0.25
	Gel electrophoresis and imaging equipment						
*TaqMan*-qPCR	Real-time PCR machine	-	1 h 45 min	1	2 PCR primers	2	$0.70
					3 fluorescently labelled probes		
AllGlo-qPCR	Real-time PCR machine	-	1 h 45 min	1	2 PCR primers	1	$0.45
					3 fluorescently labelled probes		

*:Refers to the average cost to run one sample; the cost of the equipment was not included.

### High *kdr*mutation frequencies were detected in mosquitoes collected from central China

The *kdr* mutation frequencies in *An. sinensis* samples collected from central China (Henan, Hubei, Zhejiang, Shandong and Jiangsu) were detected using the established AllGlo-qPCR method (Figure 1, Table 3). Mosquitoes from most of the provinces in central China exhibited high *kdr* mutation frequencies (from 86.57% in Jiangsu to 97.71% in Zhejiang), with the exception of Hubei, where the mutation frequency was relatively low (45.38%). Overall, 321 out of 337 individual *An. sinensis* mosquitoes (95.25%) carried at least one copy of the TTT mutated *kdr* allele.

**Table 3:** ***Kdr*** **mutation frequencies in** ***An. sinensis*** **samples from central China, as detected by AllGlo-qPCR**

Field site	Sample size	Genotype						Mutation frequency
		TTG/TTG	TTT/TTT	TGT/TGT	TTG/TTT	TTG/TGT	TTT/TGT	
Henan	42	1	18	7	6	0	10	90.48%
Hubei	48	22	9	3	7	0	7	45.38%
Zhejiang	131	1	105	3	4	0	18	97.71%
Shandong	48	4	28	1	1	0	14	90.63%
Jiangsu	108	12	61	1	3	1	30	86.57%
Total	377	40	221	15	21	1	79	86.47%

## Discussion

*Kdr* mutations are strongly associated with resistance to insecticides, especially pyrethroids and DDTs, in many malaria vectors. Previous studies have focused on screening for *kdr* mutations in *Anopheles* mosquitoes. Currently, a number of assays are available for genotyping *kdr* alleles, including AS-PCR [13], heated oligonucleotide ligation assay (HOLA), sequence-specific oligonucleotide probe enzyme-linked immunosorbent assay (SSOP-ELISA) [16], PCR-Dot, *TaqMan* probe-based analyses, high-resolution melt (HRM) analysis [17, 18], rPASA, fluorescence resonance energy transfer/melting curve analysis (FRET/MCA) [19], PCR extension with fluorescence [20], allele-specific loop-mediated isothermal amplification (AS-LAMP) [21], and more. AS-PCR is the method that is most widely used in countries that are endemic for malaria, most likely due to its relatively low cost (in terms of the equipment needed and cost per run). However, the reliability of this technique is easily affected by the accuracy of primer design, optimization of the reaction system and the difficulty in distinguishing ambiguous bands, which limits its application. In recent years, more scientists have turned to real-time PCR-based assays to detect *kdr* mutations, due to their ease of use and higher reliability [17, 22].

This report is the first to describe the development of an AllGlo-qPCR assay for detecting *kdr* mutations in *Anopheles* mosquitoes. Unlike traditional design real-time probes such as *TaqMan* probes, which have a fluorophore at the 5’ end and a nonfluorescent quencher at the 3’ end, AllGlo probes have two identical reporter dyes that normally quench themselves. Upon hybridization with the target sequence, the labelled oligo becomes stretched and cleaved, leading to separation of the two reporter dyes and consequent fluorescence. Therefore, these probes offer much higher sensitivity than traditional *TaqMan* probes. This general observation is supported by the results from this study, which show that the AllGlo-qPCR method has greater sensitivity than the AS-PCR and *TaqMan*-MGB methods. Out of the 158 samples tested, the AllGlo-qPCR method generated only two incorrect genotyping results, which might have been due to poor DNA extraction and reaction conditions. Another advantage of AllGlo-qPCR is that it is less expensive than *TaqMan*-MGB. While the *kdr* wild-type genotype and both mutations are detected in a single reaction tube in AllGlo-qPCR, two independent reaction tubes are required for the detection of these three *kdr* alleles by *TaqMan*-qPCR. The use of fewer probes and reaction reagents and a simpler probe design contribute to the reduced cost of AllGlo-qPCR (Table 2). In addition, the clustering of samples in scatter plots leads to simple and high-throughput genotype scoring for detecting *kdr* mutations. Therefore, use of the AllGlo-qPCR method should be considered in areas of malaria transmission to screen for *kdr* mutations in *An. sinensis* and other malaria vectors, as long as resources exist for purchasing and maintaining a real-time PCR machine.

The *kdr* mutations were successfully detected by AllGlo-qPCR in *An. sinensis* samples collected from the other four provinces in central China, suggesting the wild *An. sinensis* mosquitoes in these regions share similar *kdr* mutations as those in Jiangsu Province. The predominant *kdr* allele detected was L1014 F (TTT), with a small percentage of L1014C (TGT) alleles, which is consistent with previous studies of *An. sinensis* and other mosquito species, e.g., *Culex pipiens pallens* [7, 23], indicating a similar genetic outcome under selective pressure from insecticide treatment. The high frequency of *kdr* mutation (more than 87%) observed in this study from samples collected in central China is consistent with the other studies of *An. sinensis* [24, 25]. A high level of resistance to insecticides (mainly the pyrethroids) has been reported in wild *An. sinensis* in central China, and, according to previous studies, most of the regions with high insecticide resistance levels have high *kdr* mutation frequencies [7, 24, 26]. However, an exception to this rule was found in the Hubei province, which has a high percentage of resistance in the wild *An. sinensis* population, though a relatively low frequency of *kdr* mutation (45.38%) was detected. In 2011, a higher frequency of *kdr* mutation (94.8% of 122 wild samples) was detected in Wuxue, Hubei, an area of where many mosquitoes exhibit deltamethrin resistance [7], suggesting that the frequency of *kdr* mutation differs significantly according to the mosquito collection site, even in areas with similar geographical characteristics [27]. Another possible explanation is that other factors could also be involved in the insecticide resistance. For example, insecticide resistance levels were recently found to continue to increase during the pyrethroid selection process even when the *kdr* mutation frequency reaches a maximum (100%) in a wild *An. sinensis* population (unpublished data). These observations are consistent with a recent study conducted in Anhui (Figure 1), where the authors concluded that metabolic detoxification, and not the L1014 *kdr* mutation, may be the dominant mechanism of insecticide resistance in *An. sinensis* in this region [28], suggesting a complex mechanism for insecticide resistance in *An. sinensis*. However, identifying the mechanism of insecticide resistance in *An. sinensis* is outside of the scope of the present study, as the main purpose was to establish the novel AllGlo-qPCR method for detecting *kdr* mutations.

## Conclusion

This report is the first to describe a high-throughput AllGlo-qPCR assay that can be used to detect *kdr* mutations in *Anopheles* mosquitoes. Compared to two other previously reported methods, the AllGlo-qPCR method delivers the greatest specificity and sensitivity at a reasonable cost per run. This assay could be widely used to screen for *kdr* mutations in *An. sinensis* in central China, and it has the potential to be used for other mosquito species in regions of malaria transmission.
